# The Potential Mediating Role of Inflammation on the Association Between Dietary Inflammatory Index and Sleep Disturbance Among Breast Cancer Patients: A Cross-Sectional Study

**DOI:** 10.3390/nu17243889

**Published:** 2025-12-12

**Authors:** Zhenzhen Huang, Lan Cheng, Jianyun He, Xinxin Cheng, Yuting Wang, Xiaoxia Lin, Xinyi Miao, Ran Wang, Shufang Xia

**Affiliations:** Wuxi School of Medicine, Jiangnan University, Wuxi 214122, China; huangzhenzhen@stu.jiangnan.edu.cn (Z.H.); chenglan@stu.jiangnan.edu.cn (L.C.); hejianyun@stu.jiangnan.edu.cn (J.H.); chengxinxin@stu.jiangnan.edu.cn (X.C.); wangyuting@stu.jiangnan.edu.cn (Y.W.); linxiaoxia@stu.jiangnan.edu.cn (X.L.); miaoxinyi@stu.jiangnan.edu.cn (X.M.); wangran@stu.jiangnan.edu.cn (R.W.)

**Keywords:** breast cancer, sleep disturbance, dietary inflammatory index, inflammation, nutrients

## Abstract

**Background**: Sleep disturbance (SD) is very common in breast cancer (BC) patients, resulting in poor therapeutic efficacy and prognosis. Diet may be associated with SD through systemic inflammation. This study aimed to evaluate the association between the energy-adjusted Dietary Inflammatory Index (E-DII) and SD, as well as the potential mediating role of inflammatory biomarkers in patients with BC. **Methods**: In this cross-sectional study, 302 BC patients were recruited, from whom 103 blood samples were obtained for the determination of plasma inflammatory biomarkers. Dietary intake was evaluated using 3-day, 24 h dietary recalls, while SD was assessed using the Pittsburgh Sleep Quality Index (PSQI). **Results**: SD was observed in 91 (30.13%) patients, who exhibited significantly higher E-DII scores, C-reactive protein (CRP), interleukins (IL-1β, IL-6, and IL-10), and tumor necrosis factor-α (TNF-α) levels compared to non-SD participants (*p* < 0.05). After adjusting for covariates, for every 1-point elevation in E-DII, the risk of SD increased by 23.0% (OR = 1.23; 95% CI: 1.04, 1.44; *p* = 0.014). Among the E-DII components, only vitamin C showed an inverse correlation with SD (OR = 0.99; 95% CI: 0.99, 1.00; *p* = 0.015). Mediation analysis showed that IL-1β, IL-10, IL-6, TNF-α, and CRP statistically mediated the association between E-DII and SD (all *p* < 0.05). The sensitivity parameters ρ were 0.3, 0.5, 0.4, 0.4, and 0.4, respectively. **Conclusions**: A diet with pro-inflammatory potential was correlated with SD among BC patients, which might be mediated by circulating IL-1β, IL-10, IL-6, TNF-α, and CRP.

## 1. Introduction

Breast cancer (BC) is a major health concern for women in China, with its incidence ranking second and its mortality ranking fifth among female malignancies [1]. From the time of disease diagnosis, during treatment, and throughout survivorship, sleep disturbance (SD) emerges as a prevalent symptom frequently reported by BC patients, with prevalences ranging from 14% to 93% [2]. However, it is often seen as a temporary secondary symptom of BC treatments and neglected by clinicians [3]. As a result of untreated SD, patients with BC are particularly vulnerable to fatigue, immune compromise, and cognitive impairment, which can even lead to BC recurrence and increased mortality [4]. Given the overall high prevalence and the numerous adverse effects of SD, identifying modifiable risk factors for potentially effective interventions is crucial for enhancing the overall health status and prognosis of patients with BC.

As an essential and modifiable behavioral determinant for human health, diet has been increasingly recognized for its association with sleep. Recent findings indicated that adherence to balanced and healthy dietary patterns—characterized by higher consumption of fiber, fruits, vegetables, and anti-inflammatory nutrients and lower intake of saturated fat (e.g., the Mediterranean diet)—was associated with better sleep quality [5]. In contrast, unhealthy foods such as cured meat and added sugars are linked to an increased risk for SD [6]. The deficiency of certain nutrients in food, such as polyunsaturated fatty acids (PUFAs) and vitamins, can also lead to poorer sleep outcomes [7]. Growing evidence has shown that the mechanisms responsible for the role of diet on sleep might include inflammation [8], the microbiota-gut–brain axis [9], and oxidative stress [10]. Among them, inflammation has attracted significant attention from researchers. The Mediterranean diet, with an increased intake of fruits, vegetables, and fish, and a moderate intake of red and processed meats, has been shown to reduce systemic inflammation [11], and high adherence to this diet led to improved sleep quality [12]. Vegetables, fruits, and fish are good sources of dietary fiber, vitamins, polyphenols, and PUFAs, which can help reduce the levels of inflammatory biomarkers such as interleukins (IL-1β and IL-6) and C-reactive protein (CRP) [9,13,14]. Conversely, excessive intake of red meat or processed meat promotes inflammation by up-regulating cytokine production [15]. The aforementioned inflammatory biomarkers were all reported to be critical in sleep. For instance, a longitudinal study illustrated that increased sleep duration or decreased SD were associated with the attenuation of pro-inflammatory biomarkers (such as IL-1β and IL-6) and the counter-regulatory cytokine (IL-10) among cancer survivors [16]. Given the importance of diet in regulating systemic inflammation, researchers developed the dietary inflammatory index (DII) and the energy-adjusted DII (E-DII) to assess the overall inflammatory potential of an individual’s diet [17]. These two indices have been shown to be correlated with poor sleep outcomes in the general population [18], but limited research has investigated the influence of dietary inflammatory potential on inflammation and SD among BC patients.

Therefore, in this study, we hypothesized that diets with higher pro-inflammatory potential might correlate with SD in patients with BC, in which inflammation might play a role. The objectives were to determine the prevalence of SD and the dietary nutritional status among BC patients, and to establish the connection between the E-DII-based diet and SD while exploring the potential mediating effect of inflammatory biomarkers.

## 2. Materials and Methods

### 2.1. Participants and Study Design

A total of 302 BC patients were enrolled in this cross-sectional study conducted at the Affiliated Hospital of Jiangnan University, spanning from August 2023 to April 2024. The inclusion criteria were: female BC without metastasis and diffusion; aged ≥18 years; hospitalization for postoperative chemotherapy; normal cognitive function and reading ability; and voluntary participation in this research. Exclusion criteria included: diagnosed with other cancers or terminal diseases; diagnosed with gastrointestinal, hematological, immune, or inflammatory diseases; receiving targeted therapy, radiotherapy; use of sleep aids, anxiolytics, antidepressants, antibiotics, or other medications that affect immunity in the past three weeks; and incomplete questionnaires or clinical data. This study received approval from the Medical Ethics Committee of Jiangnan University (JNU20230601IRB10) and was registered at the Chinese Clinical Trial Registry (ChiCTR2300074687). The study was conducted in accordance with the Declaration of Helsinki (1989), and all participants submitted written informed consent.

### 2.2. Sample Size

Sample size estimation was based on the Monte Carlo simulation results reported by Fritz et al. [19]. In a single-mediator model, the path from the independent variable to the mediator was denoted as *a*, and the path from the mediator to the dependent variable as *b*. The indirect effect was tested using the bias-corrected bootstrap method with 10,000 resamples. When both *a* and *b* had standardized coefficients representing medium effect sizes (0.39) [20], a sample size of 71 was required to achieve 0.80 power at α = 0.05 (two-tailed). Accounting for an estimated 20% dropout, a total of 86 BC patients undergoing chemotherapy were planned for recruitment to ensure that the power of the indirect effect test for each mediator model would be ≥0.80.

### 2.3. Assessment of SD

The Chinese version of the Pittsburgh Sleep Quality Index (PSQI) was applied to assess participants’ sleep status during the previous month [21]. The instrument includes 19 self-rated items that are summarized into seven dimensions: perceived sleep quality, sleep latency, sleep duration, habitual sleep efficiency, sleep disturbances, use of sleep medications, and daytime dysfunction. Each dimension is scored from 0 (indicating better sleep) to 3 (indicating poorer sleep), yielding a global score ranging from 0 to 21. In this study, individuals with a total PSQI score greater than or equal to 8 were categorized as SD patients, whereas those scoring below 8 were classified as non-SD patients [22].

### 2.4. Dietary Intake Assessment and E-DII Calculation

Dietary intake was assessed using three non-consecutive 24 h dietary recalls. The first recall was conducted on the day of hospital admission, capturing dietary intake from the previous day. The subsequent two recalls were performed during the recovery period after discharge (one on a weekday and one on the weekend). The post-discharge recalls were completed via WeChat or telephone after the resolution of chemotherapy-related gastrointestinal symptoms. Each recall collected detailed information on food types, portion sizes, and cooking methods. During the dietary survey, food models and atlases were utilized to facilitate dietary recall. Moreover, in order to improve the accuracy of the dietary survey, the patients’ caregivers were encouraged to assist in the recall process. For incomplete or partially missing dietary recalls, participants were contacted via telephone, WeChat, or in person to obtain information on the missing food types, portion sizes, and cooking methods. Participants whose dietary data were temporarily unavailable were invited to complete the dietary assessment at a later date to ensure valid data collection. The collected dietary data were then entered into the Nutrition Calculator v2.8.3.0 (Beijing, China) to obtain the average daily intake of nutrients. The software draws its ingredient data from the Sixth Edition of the China Food Composition Tables, which represents the most widely recognized and rigorously reviewed compilation of food nutritional data in China. The intake of β-carotene, saturated fatty acids (SFA), monounsaturated fatty acids (MUFA), PUFA, omega-3 and omega-6 fatty acids was manually calculated based on the Sixth Edition of the China Food Composition Tables, as these nutrients are not included in the Nutrition Calculator v2.8.3.0.

DII is an algorithm created to assess the dietary inflammatory potential, derived from 45 food parameters encompassing micronutrients, macronutrients, and individual food items [23]. Because certain DII components were rarely consumed by Chinese BC patients and the incomplete database for the calculation of some food parameters, a total of 25 dietary nutrients (including energy, carbohydrates, fat, protein, dietary fiber, vitamins A, B1, B2, B6, C, D, E, folate, niacin, β-carotene, cholesterol, iron, zinc, magnesium, selenium, SFAs, MUFAs, PUFAs, omega-3, and omega-6 fatty acids) were finally included for E-DII calculation. Previous work has calculated the DII based on 25 food parameters derived from the predefined list of 45 food parameters, and this approach has been commonly adopted [24,25,26]. To adjust for the effect of different total energy intakes on the DII score, the E-DII was computed using established methodologies documented in prior literature [27]. Elevated E-DII scores indicate higher pro-inflammatory potential, whereas lower scores suggest stronger anti-inflammatory potential. The participants were stratified into three groups based on E-DII tertiles: T1 group (−4.40, −0.90), T2 group (−0.89, 0.88), and T3 group (0.89, 4.47).

### 2.5. Inflammatory Biomarkers Determination

Fasting venous blood samples were voluntarily provided by 103 participants prior to chemotherapy for the assessment inflammatory biomarkers. Blood was centrifuged at 3500 rpm for 10 min to obtain plasma. IL-1β, IL-10, IL-6, tumor necrosis factor α (TNF-α), and CRP were tested using commercially available ELISA kits (Bioswamp Life Science Lab, Wuhan, China) in accordance with the manufacturer’s protocols. According to the kit specifications, the intra-assay and inter-assay coefficients of variation (CVs) were both <10%, indicating acceptable analytical precision. All plasma samples were measured in triplicate. The majority of sample-specific CVs were <10%, with a small proportion ranging from 10% to 15%, all within acceptable limits. Quality control procedures included routine use of standard curves, blanks, positive controls, and randomization of sample order to minimize batch and plate-position effects. These procedures ensured high assay reproducibility and minimized potential measurement error or attenuation bias.

### 2.6. Other Variables

Physical activity level was evaluated using the International Physical Activity Questionnaire Short Form (IPAQ-SF) to determine the metabolic equivalent (MET) and then categorized into three levels: low (<600 MET/min/week), moderate (600 to 1500 MET/min/week), and high (>1500 MET/min/week) [28]. The Visual Analogue Scale (VAS) was used to assess the pain status of the patients [29], and the psychological status, including anxiety and depression, was evaluated using the Hospital Anxiety and Depression Scale (HADS) [30]. The HADS consists of an anxiety subscale (7 items, HADS-A) and a depression subscale (7 items, HADS-D).

### 2.7. Statistical Analysis

Statistical analysis was conducted using SPSS 27.0. The Kolmogorov–Smirnov test was used to evaluate the normality of continuous variables. Normally distributed data were presented as mean ± standard deviation and compared by independent sample *t*-test or one-way ANOVA. Non-normally distributed data were presented as median (25th, 75th percentile) and compared by the Mann–Whitney U test or the Kruskal–Wallis test. Categorical variables were expressed as frequencies (*n*) and percentages (%) and were compared using the Chi-square test or continuity-corrected Chi-square test. Logistic regression analysis was used to analyze the associations between E-DII, its food parameters, or inflammatory biomarkers, with SD. Linear regression analysis was conducted to assess the relationships between E-DII and its food parameters with inflammatory biomarkers. Trend test was performed by assigning the median value of each tertile as a continuous variable to determine if the odds of SD increased across the tertiles of the E-DII. Since values of plasma inflammatory markers are typically right-skewed, they were log-transformed before subsequent statistical analyses. R Mediation package 4.5.0 was used to explore the potential mediating effects of inflammatory biomarkers on the association between E-DII and SD (Figure S1). We computed the average causal mediation effect (ACME, the statistical indirect effect of E-DII on SD through the inflammatory biomarkers), the average direct effect (ADE, the statistical direct effect of E-DII on SD while excluding inflammatory biomarkers), and the average total effect (ATE, the sum of ACME and ADE). The 95% confidence interval (CI) was estimated by nonparametric bootstrapping with 5000 replications. To evaluate the potential impact of unmeasured confounding on the mediation effect, we conducted a parametric sensitivity analysis, denoting the correlation between the residuals of the mediator and outcome models as ρ. In the mediation R package (v.4.5.0), the sensitivity parameter is evaluated over a default grid from −0.9 to 0.9 in increments of 0.1 [31]. When no unmeasured confounding is present, ρ = 0, whereas ρ ≠ 0 indicates the presence of unmeasured confounding. Larger critical values of ρ (closer to ±1) imply that stronger confounding would be required to invalidate the observed mediation effect, indicating greater robustness. Conversely, smaller critical ρ values (close to 0) suggest that even mild confounding could overturn the results, indicating weaker robustness [31]. Additionally, to ensure the robustness of the logistic regression estimates, all logistic regression models underwent diagnostic checks. Linearity in the logit for continuous predictors was assessed using the Box–Tidwell test, potential influential observations and high-leverage points were examined via Cook’s distance and leverage values, and residual normality was evaluated using standardized residuals. Multicollinearity was assessed using variance inflation factors (VIF), with values < 5 considered indicative of no serious multicollinearity.

## 3. Results

### 3.1. Participants’ Characteristics

Among the 302 BC patients, 91 (30.13%) individuals with PSQI scores ≥ 8 were categorized into the SD group, while the remaining 211 patients (69.87%) were placed in the non-SD group. In Table 1, statistical significances were detected between the two groups in education level (*p* = 0.021), family monthly income (*p* < 0.001), pain score (*p* = 0.006), anxiety score (*p* < 0.001), and depression score (*p* < 0.001).

### 3.2. Nutrient Intakes in Different E-DII Tertiles

As shown in Table S1, the consumption of energy, protein, fat, dietary fiber, vitamins (A, B2, B6, C, D, E, and folate), β-carotene, magnesium, iron, selenium, zinc, SFAs, MUFAs, PUFAs, and omega-6 fatty acids was significantly different among the E-DII tertiles (*p* < 0.05).

### 3.3. Nutrient Intake of SD and Non-SD Patients

Table 2 displays the differences in nutrient intake between BC patients with SD and those with non-SD. Patients with SD exhibited significantly higher E-DII scores (*p* = 0.030) and a higher proportion of participants consuming pro-inflammatory diets (*p* = 0.010) compared to non-SD patients. Additionally, SD patients had significantly lower consumption of dietary fiber, folate, vitamin C, niacin, zinc, and magnesium (*p* < 0.05).

### 3.4. Association Between E-DII and SD

When the PSQI score was converted into a dichotomous variable, and after adjusting for age, BMI, physical activity level, education level, family monthly income, cancer stage, pain score, anxiety and depression score, patients in the highest E-DII tertile had a 2.34-fold increased risk of SD compared to those in the lowest E-DII tertile (Table 3, OR = 2.34; 95% CI: 1.18, 4.62; *p* for trend = 0.031). In the adjusted model, when E-DII was treated as a continuous variable, every 1-point increase in E-DII was associated with a 23.0% higher risk of SD in BC patients (Table 4, OR = 1.23; 95% CI: 1.04, 1.44; *p* = 0.014). Furthermore, among all E-DII components, only vitamin C remained significantly correlated with SD in BC patients after adjustment (OR = 0.99; 95% CI: 0.99, 1.00; *p* = 0.015).

The linearity assumption of continuous variables in the logit was not violated, as the test for linearity was non-significant, indicating that the assumption was met. No influential or high-leverage observations were identified based on Cook’s distance and leverage values, further supporting robustness of the model fit (Table S2). In both fully adjusted categorical and continuous E-DII logistic regression models, all VIF values ranged from 1.015 to 1.934, which is well below the threshold of 5, indicating no evidence of multicollinearity (Tables S3 and S4).

When the PSQI score was treated as a continuous variable, it was log-transformed due to its non-normal distribution, and linear regression was used to analyze its association with both continuous and categorical E-DII. No significant linear association between E-DII and the PSQI score was observed (Table S5, all *p* > 0.05).

### 3.5. Plasma Inflammatory Biomarkers in SD and Non-SD Patients

To assess potential non-response bias, we compared the demographic characteristics, clinical variables, and E-DII between participants who provided blood samples for inflammatory biomarker analysis (*n* = 103) and those who did not provide blood samples (*n* = 199). As shown in Table S6, no significant differences were observed between the two groups on the aforementioned variables (all *p* > 0.05).

**Figure 1:**
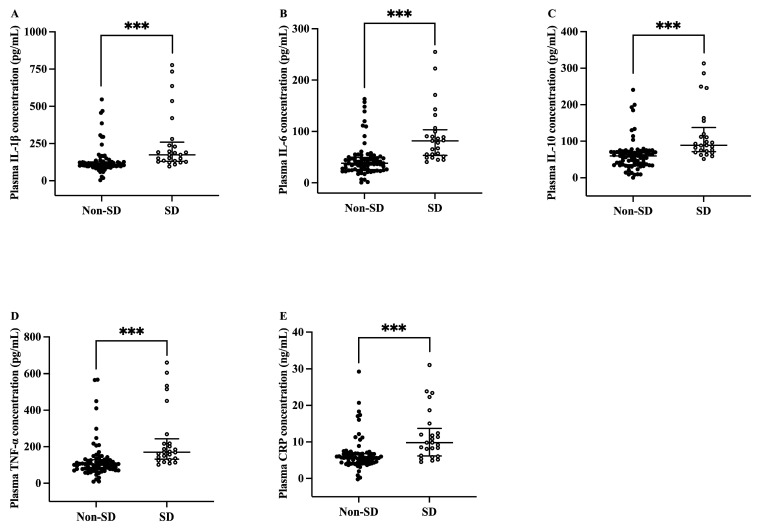
Plasma inflammatory biomarker levels of sleep disturbance and non-sleep disturbance patients with breast cancer (*n* = 103). (**A**) IL-1β; (**B**) IL-6; (**C**) IL-10; (**D**) TNF-α; (**E**) CRP. Data were shown as median (25th, 75th percentile). Mann–Whitney U test was used. *** *p* < 0.001. CRP, C-reactive protein; IL-1β, interleukin 1β; IL-6, interleukin 6; IL-10, interleukin 10; SD, sleep disturbance; TNF-α, tumor necrosis factor α.

As shown in Figure 1A–E, patients with SD demonstrated remarkably increased plasma IL-1β, IL-6, IL-10, TNF-α (measured in pg/mL), and CRP (measured in ng/mL) levels than the non-SD patients (*p* < 0.001).

### 3.6. Association Between E-DII and Plasma Inflammatory Biomarkers

As demonstrated in Table 5, higher E-DII scores were significantly associated with elevated levels of IL-1β, IL-6, IL-10, TNF-α, and CRP according to multiple linear regression analysis (*p* < 0.05).

### 3.7. Association Between Plasma Inflammatory Biomarkers and SD

After adjusting for the covariates, IL-1β, IL-6, IL-10, TNF-α, and CRP were all positively correlated with SD in patients with BC (*p* < 0.001, Table 6).

### 3.8. Association Between E-DII and SD with Inflammatory Biomarkers as Mediators

The mediation analysis (Table 7) showed that all five biomarkers demonstrated statistically significant indirect effects on the association between E-DII and SD. Specifically, the ACME values were 0.031 for IL-1β (95% CI: 0.007–0.066; *p* = 0.004), 0.027 for IL-6 (95% CI: 0.001–0.062; *p* = 0.039), 0.048 for IL-10 (95% CI: 0.011–0.096; *p* = 0.004), 0.028 for TNF-α (95% CI: 0.007–0.063; *p* = 0.004), and 0.024 for CRP (95% CI: 0.002–0.057; *p* = 0.032). Sensitivity analysis indicated that the sensitivity parameters (ρ) for these mediators ranged from 0.3 to 0.5.

## 4. Discussion

In the present study, we found that BC patients who consumed a pro-inflammatory diet with higher E-DII scores had an elevated risk of SD. Patients with SD were often accompanied by lower intakes of specific E-DII components such as vitamin C, folate, niacin, and zinc, but only vitamin C intake showed a negative correlation with SD. Of particular importance, a novel finding was the indirect statistical association of IL-1β, IL-6, IL-10, TNF-α, and CRP on the relationship between E-DII and SD, suggesting that inflammation may play a role in the observed link between diet and SD in BC patients.

SD has emerged as a prevalent symptom among patients with BC. We found that 30.13% of patients with BC experienced SD, which was slightly lower than the prevalence reported in previously published literature [3]. One reason for this discrepancy could be the use of a PSQI score ≥ 8 as the cutoff for SD in this study, whereas previous studies typically used a score of 5 as the cutoff [3]. We chose this cutoff of 8 scores based on the study that suggested it was more appropriate for screening SD in the Chinese cancer population [22]. Another reason might be that we excluded patients using sleep aids (e.g., melatonin) when recruiting participants, as these medications could affect appetite, sleep, and inflammation [32], and these excluded patients were likely to have SD already. In this study, we also found that patients with SD had a higher proportion of participants with lower educational attainment and family monthly incomes. In general, patients with higher education and greater economic assistance in the Chinese population are more likely to obtain good jobs, have more wealth accumulation, enhance disease awareness and capacity for optimal health decision-making, and elevate psychological resilience (e.g., less death anxiety), ultimately resulting in good sleep quality [33,34]. A meta-analysis has suggested that anxiety, depression, and pain were all significantly associated with SD in BC patients [2]. Our study also found similar results. Poor pain management had a long-term impact on sleep quality in BC patients, which could further exacerbate symptoms of anxiety and depression [35]. Therefore, healthcare professionals should be concerned about BC patients with lower education or income, higher levels of pain, anxiety, or depression, and take targeted measures to prevent or improve SD, thereby preventing the occurrence of multiple complications.

The beneficial effects of dietary nutrition on the treatment and prognosis of BC patients are increasingly recognized by researchers [36]. In the present study, we found that a diet with pro-inflammatory potential was consistently correlated with SD, indicating an indirect association between dietary inflammatory potential and sleep among patients with BC. These findings were consistent with those of the general population. For example, some studies suggest that adults who consume diets rich in pro-inflammatory foods are more susceptible to experiencing adverse sleep outcomes, including prolonged or insufficient sleep durations, self-reported SD, and poor sleep quality [37,38]. Similarly, in overweight or obese pregnant women, pro-inflammatory diets had correlations with longer sleep latency and wake-after-sleep-onset [39]. However, among patients with sleep apnea, similar associations between DII scores and sleep parameters could not be found [40]. Interestingly, police officers whose diets with higher pro-inflammatory potential even had improved subjective sleep quality [41]. A systematic review involving cohort study, cross-sectional study, and intervention study has suggested that there was no significant association between DII and overall sleep quality, sleep duration, and other sleep metrics based on the majority of the evidence [42]. Overall, diets with pro-inflammatory potential were related to poor sleep in at least one sleep domain [43], but there was heterogeneity across populations and sleep metrics. The main reasons for this heterogeneity might include differences in sleep assessment methodology (e.g., subjective versus objective measurements), the variety of study populations, and the lack of adjustment for important but unidentified covariates, making it fundamentally difficult to compare results from different studies [43]. In addition, no significant linear association was observed between E-DII and continuous PSQI scores. This discrepancy may be explained by several factors. First, when PSQI is treated as a continuous variable, scores may exhibit substantial individual variability, non-normality, or high variance, making subtle associations difficult to detect using linear regression. Second, continuous analyses typically require larger effect sizes, and the relatively small sample size in this study may have limited the ability to achieve statistical significance. Therefore, well-designed large-scale longitudinal studies and strictly controlled clinical trials with rigorous sleep assessments (e.g., polysomnography) and more potentially important covariates considered are needed to confirm the influence of diet on SD or other sleep symptoms in different populations, especially in patients with BC who are more sensitive to dietary inflammatory potential.

Studies in humans and animal models are beginning to unravel the underpinnings of the association between diet and SD, of which inflammation is believed to play an essential role. On the one hand, epidemiological studies have indicated that the E-DII score could predict concentrations of inflammatory biomarkers [44,45]. The EPIC cohort study demonstrated that E-DII and DII scores were positively correlated with elevated CRP, TNF-α, and IL-6 levels in 17,637 participants [46]. A systematic review has also revealed that the anti-inflammatory diet could reduce IL-1β, IL-6, and CRP production and increase IL-4, IL-10, and IL-13 to inhibit inflammation in adult human populations [47]. Here, we also observed that E-DII scores were positively correlated with IL-1β, IL-6, IL-10, TNF-α, and CRP levels in patients with BC. Surprisingly, primarily acting as a major anti-inflammatory biomarker, IL-10 also had pro-inflammatory effects and was associated with the pro-inflammatory diet, which might be attributed to the body’s negative feedback in the inflammatory response by increasing its concentration to counteract inflammation. On the other hand, growing evidence has illustrated that inflammation might play an important role in SD. A cross-sectional study based on data from NHANES 2015–2020 found that elevated levels of various inflammatory markers, such as CRP, were significantly positively associated with the occurrence of SD [48]. Among cancer patients, sleep similarly exhibits a strong inflammatory basis [49]. A longitudinal study of 71 cancer survivors suggested that attenuation in IL-1β, IL-6, TNF-α, and IL-10 was associated with either an increased sleep duration or decreased sleep problems [16]. Another longitudinal study of 53 patients with BC undergoing chemotherapy demonstrated similar positive correlations between changes in PSQI-assessed sleep quality and IL-6 and IL-1 receptor antagonists, as well as between total nighttime awake time and CRP [50]. In accordance with these findings, our results also showed that IL-1β, IL-10, IL-6, CRP, and TNF-α were all positively associated with SD. Given that all inflammatory biomarkers measured were remarkably correlated with SD and E-DII, we further performed a mediation analysis and found that all five inflammatory biomarkers (IL-6, IL-1β, IL-10, TNF-α, and CRP) showed statistically significant mediation effects on the association between E-DII and SD, suggesting the potential roles of inflammatory biomarkers in the association between pro-inflammatory diets and SD. Similar findings have also been reported in non-cancer populations. A study based on the UK Biobank demonstrated that healthier dietary patterns were significantly associated with better sleep quality, and this relationship was largely mediated by inflammatory markers such as platelet count, neutrophil to lymphocyte ratio, and CRP [8]. In our sensitivity analyses, the critical ρ values required to reduce the ACME to zero ranged from 0.3 to 0.5 for IL-1β, IL-10, IL-6, TNF-α, and CRP. This indicates that a non-trivial degree of unmeasured confounding would be necessary to fully eliminate the observed mediation effects, suggesting a certain level of robustness. Nonetheless, the potential influence of mild to moderate unmeasured confounding cannot be entirely ruled out, and the robustness of our effect estimates remains limited. Therefore, caution is warranted when interpreting the mediation results. Furthermore, due to the cross-sectional nature of the study, the observed indirect effects reflect statistical associations rather than strict causal relationships. Future studies should employ more rigorous confounding control or longitudinal designs to further investigate the role of inflammatory biomarkers in the association between E-DII and SD in BC patients.

Regarding the E-DII components, it is noteworthy that the nutrient intake of BC patients in our study was significantly lower than the recommended nutrient intake (RNI) for women of the same age group in China, particularly for energy and certain vitamins, such as folate and vitamin B6. These results are consistent with a cohort study conducted in Hong Kong, which reported that BC patients’ nutrient intake remained suboptimal even 3 years after diagnosis [51]. Specifically, the average energy intake of BC patients in our research was similar to that observed in the Hong Kong cohort at 1.5 and 3 years post-diagnosis (approximately 1300 kcal/day). However, folate intake in our study was higher than that reported in the Hong Kong study, vitamin B6 intake was lower. One possible explanation for these discrepancies is the changes in dietary patterns that occur after BC diagnosis. Patients with BC exhibited insufficient attention to the intake of fruits and vegetables, leading to lower levels of vitamin consumption. It is well-documented that cancer patients often modify their dietary habits, either due to treatment side effects, changes in appetite, or psychological factors [52]. For instance, many patients adopt restrictive dietary practices based on misconceptions or fear of cancer recurrence. In our study, a significant number of patients avoided certain foods, such as poultry and seafood, believing these might contribute to cancer recurrence, while simultaneously overemphasizing protein intake from foods like eggs and red meat. This dietary imbalance resulted in excessive cholesterol and iron intake, contributing to higher-than-recommended levels of these nutrients. The imbalance in the diets of BC patients highlights the importance of incorporating nutritional education into clinical practice.

In our study, compared with Non-SD patients with BC, patients with SD exhibited lower consumption of anti-inflammatory nutrients, such as dietary fiber, vitamin C, folate, niacin, magnesium, and zinc. After adjusting for covariates, only vitamin C remained significantly associated with SD. This finding was consistent with what was reported by Grandner in a study of 4548 adults, which indicated that vitamin C showed an independent correlation with non-restorative sleep [53]. As an essential micronutrient with powerful anti-inflammatory and antioxidant properties, vitamin C was capable of augmenting the effectiveness of pharmacological treatments and reducing their adverse effects in BC patients [54], which probably thereby influenced the occurrence and progression of SD. Recent research has shown that serum vitamin C levels were significantly negatively associated with trouble sleeping, with a protective effect observed in females, but no significant association found in males [55]. Combined with the fact that most patients with BC are women, it is possible that female patients may benefit more from consuming foods rich in vitamin C to improve their sleep problems. Notably, after adjusting for covariates, our study found that most of the E-DII components were not related to SD, whereas the E-DII score was still correlated with SD. These findings implied that dietary recommendations based on E-DII may have a stronger potential to regulate SD in BC patients compared to simply advising changes in a single nutrient. Therefore, healthcare professionals can utilize E-DII-based dietary recommendations to encourage BC patients to eat more foods with anti-inflammatory potential, such as vegetables and fruits, while restricting or removing foods with pro-inflammatory potential, such as fried foods, sugary foods, and processed meats, as a strategy to prevent or alleviate SD.

This study was the first to explore the association between dietary inflammatory potential and SD in BC patients, as well as the possible role of biomarkers involved. However, several limitations should be acknowledged. First, it should be noted that certain E-DII components (e.g., thyme, oregano, rosemary) are rarely consumed by the Chinese population, which presents a challenge for cross-regional comparisons. Second, the cross-sectional design of this study prevents the determination of the temporal sequence between exposure and outcome, making it difficult to establish a causal relationship between diet and SD, and allowing for the possibility of reverse causation. Third, dietary intake was assessed through self-reported data collected by 3-day, 24 h dietary recalls, potentially introducing recall bias. Fourth, the biomarker sub-sample was relatively small, limiting both the precision and generalizability of the mediation analysis. The estimated ACME values exhibited wide confidence intervals even after log-transformation, reflecting substantial statistical uncertainty. Finally, several clinical factors—such as chemotherapy, surgery, and other treatment-related variables—may affect dietary intake, inflammatory biomarkers, and SD. The limited sample size prevented us from conducting stratified analyses to account for these potential covariates, which might have introduced bias or residual confounding in the observed statistical mediation effects. Therefore, the present findings should be interpreted as exploratory rather than definitive. Future studies with larger sample sizes, multi-center recruitment, or prospective longitudinal designs will be essential to enable robust stratification, reduce uncertainty, and clarify the temporal and potential causal relationships among diet, inflammation, and SD in BC patients.

## 5. Conclusions

In conclusion, this study demonstrated a positive association between pro-inflammatory diets (indicated by elevated E-DII scores) and SD in patients with BC, and this association was statistically explained in part by IL-6, IL-1β, IL-10, TNF-α, and CRP. Therefore, adherence to an anti-inflammatory diet may be a promising preventive and therapeutic strategy by reducing inflammation and thereby improving SD, the overall health status, and the prognosis of patients with BC. Additionally, dietary counseling focused on reducing dietary pro-inflammatory potential, along with targeted sleep management strategies, could be valuable components of clinical care for BC patients, potentially enhancing quality of life and supporting long-term recovery.

## Figures and Tables

**Table 1:** Characteristics of sleep disturbance and non-sleep disturbance patients with breast cancer.

Variables	Non-SD (*n* = 211)	SD (*n* = 91)	*t*/*Z*/*χ*^2^	*p*
Age (y) ^a^	52.73 ± 11.26	54.48 ± 10.97	−1.255	0.210
BMI (kg/m^2^) ^b^	23.8 (22.0, 26.0)	24.3 (22.6, 26.0)	−0.873	0.383
Menopausal status, *n* (%) ^c^				
Post-menopausal	124 (58.8)	60 (65.9)	1.372	0.242
Pre-menopausal	87 (41.2)	31 (34.1)		
Marital status, *n* (%) ^d^				
Widowed/divorced/single	8 (3.8)	3 (3.3)	1.000	0.830
Married	203 (96.2)	88 (96.7)		
Education level, *n* (%) ^c^				
Primary school or lower	37 (17.5)	28 (30.8)	9.709	0.021
Middle school	68 (32.3)	33 (36.3)		
High school/secondary school	48 (22.7)	14 (15.3)		
Junior college or higher	58 (27.5)	16 (17.6)		
Employment, *n* (%) ^c^				
Employed	65 (30.8)	19 (20.9)	3.802	0.149
Unemployed	39 (18.5)	23 (25.3)		
Retired	107 (50.7)	49 (53.8)		
History of night shift work, *n* (%) ^c^				
No	171 (81.0)	73 (80.2)	0.028	0.868
Yes	40 (19.0)	18 (19.8)		
Residence, *n* (%) ^c^				
Rural areas	46 (21.8)	23 (25.3)	0.435	0.804
Towns	17 (8.1)	7 (7.7)		
Urban areas	148 (70.1)	61 (67.0)		
Family monthly income (CNY), *n* (%) ^c^				
<3000	15 (7.1)	15 (16.5)	17.792	<0.001
3000~5000	81 (38.4)	49 (53.8)		
>5000	115 (54.5)	27 (29.7)		
Physical activity level, *n* (%) ^c^				
Low	49 (23.2)	24 (26.4)	0.453	0.797
Moderate	153 (72.5)	64 (70.3)		
High	9 (4.3)	3 (3.3)		
Pain score ^b^	0.0 (0.0, 1.0)	0.0 (0.0, 2.0)	−2.740	0.006
Anxiety score ^b^	4.0 (2.0, 6.0)	6.0 (4.0, 8.0)	−5.293	<0.001
Depression score ^b^	2.0 (1.0, 4.0)	4.0 (2.0, 8.0)	−5.251	<0.001
Presence of comorbidities, *n* (%) ^c^				
No	146 (69.2)	57 (62.6)	1.241	0.265
Yes	65 (30.8)	34 (37.4)		
Smoking status, *n* (%) ^d^				
Never	207 (98.1)	89 (97.8)	0.000	1.000
Former/current	4 (1.9)	2 (2.2)		
Drinking status, *n* (%) ^d^				
Never	200 (94.8)	86 (94.5)	0.000	1.000
Former/current	11 (5.2)	5 (5.5)		
Tea consumption status, *n* (%) ^c^				
Never/former	186 (88.2)	79 (86.8)	0.106	0.745
Current	25 (11.8)	12 (13.2)		
Coffee consumption status, *n* (%) ^d^				
Never/former	206 (97.6)	86 (94.5)	1.086	0.297
Current	5 (2.4)	5 (5.5)		
Chemotherapy cycle, *n* (%) ^c^				
T0	48 (22.7)	20 (22.0)	2.213	0.547
T1~T2	95 (45.1)	35 (38.5)		
T3~T4	46 (21.8)	22 (24.2)		
≥T5	22 (10.4)	14 (15.3)		
Cancer stage, *n* (%) ^c^				
I	64 (30.3)	34 (37.4)	3.863	0.145
II	129 (61.2)	45 (49.5)		
III	18 (8.5)	12 (13.1)		
Surgery type, *n* (%) ^c^				
Lumpectomy	81 (38.4)	32 (35.2)	0.282	0.595
Mastectomy	130 (61.6)	59 (64.8)		
Triple-negative breast cancer, *n* (%) ^c^				
No	177 (83.9)	74 (81.3)	0.299	0.585
Yes	34 (16.1)	17 (18.7)		

Data are shown as *n* (%), median (25th and 75th percentiles), or mean ± standard deviation. BMI, body mass index; CNY, China yuan; SD, sleep disturbance. ^a^ Independent samples *t*-test. ^b^ Mann–Whitney U test. ^c^ Chi-squared test. ^d^ Chi-squared test with continuity correction.

**Table 2:** E-DII and dietary nutrient intakes of sleep disturbance and non-sleep disturbance patients with breast cancer (*n* = 302).

Variables	Non-SD (*n* = 211)	SD (*n* = 91)	*Z*/*χ*^2^	*p*
E-DII (continuous) ^a^	−0.1 (−1.5, 1.0)	0.4 (−1.3, 1.9)	−2.175	0.030
E-DII (categorical), *n* (%) ^b^				
T1 (−4.40, −0.90)	73 (34.6)	28 (30.8)	9.234	0.010
T2 (−0.89, 0.88)	79 (37.4)	22 (24.2)		
T3 (0.89, 4.47)	59 (28.0)	41 (45.0)		
Nutrients				
Energy (kcal/d) ^a^	1307.0 (1088.0, 1546.0)	1242.0 (1004.0, 1506.0)	−1.163	0.245
Protein (g/d) ^a^	62.9 (48.9, 83.9)	56.9 (42.2, 79.1)	−1.544	0.123
Total fat (g/d) ^a^	47.5 (34.1, 65.6)	45.2 (31.4, 62.7)	−1.257	0.209
Carbohydrate (g/d) ^a^	143.0 (115.3, 187.3)	144.6 (107.2, 179.6)	−0.615	0.539
Dietary fiber (g/d) ^a^	9.3 (6.4, 12.6)	7.8 (5.7, 10.4)	−2.156	0.031
Cholesterol (mg/d) ^a^	561.0 (407.0, 744.0)	603.0 (383.0, 808.0)	−0.617	0.537
Vitamin A(μgRAE/d) ^a^	430.0 (312.0, 598.0)	376.0 (293.0, 507.0)	−1.921	0.055
Vitamin B1 (mg/d) ^a^	0.6 (0.5, 0.8)	0.6 (0.5, 0.8)	−0.143	0.886
Vitamin B2 (mg/d) ^a^	1.0 (0.8, 1.3)	0.9 (0.6, 1.3)	−1.651	0.099
Vitamin B6 (mg/d) ^a^	0.2 (0.1, 0.3)	0.1 (0.1, 0.2)	−1.855	0.064
Vitamin C (mg/d) ^a^	115.7 (75.7, 171.1)	84.4 (46.0, 136.2)	−3.560	< 0.001
Vitamin D (μg/d) ^a^	1.7 (0.3, 4.5)	1.5 (0.0, 3.4)	−0.770	0.441
Vitamin E (mg/d) ^a^	16.1 (11.7, 19.9)	14.8 (10.6, 18.7)	−1.592	0.111
Folate (μg/d) ^a^	109.6 (67.1, 157.2)	81.4 (58.8, 145.8)	−2.123	0.034
Niacin (mg/d) ^a^	12.9 (9.4, 16.2)	11.0 (8.0, 14.1)	−2.495	0.013
Magnesium (mg/d) ^a^	268.0 (204.0, 331.0)	229.0 (182.0, 271.0)	−3.129	0.002
Iron (mg/d) ^a^	16.6 (13.1, 21.2)	15.1 (11.9, 19.5)	−1.810	0.070
Zinc (mg/d) ^a^	9.7 (7.5, 11.8)	8.4 (6.1, 11.2)	−2.301	0.021
Selenium (μg/d) ^a^	49.5 (32.8, 71.5)	50.1 (31.8, 63.3)	−0.493	0.622
β-Carotene (ug/d) ^a^	1423.4 (761.2, 2546.0)	1212.0 (699.4, 1975.8)	−1.130	0.259
SFAs (g/d) ^a^	13.7 (10.0, 19.2)	14.9 (8.6, 20.8)	−0.017	0.987
MUFAs (g/d) ^a^	16.6 (11.7, 23.6)	16.6 (10.5, 24.2)	−0.369	0.712
PUFAs (g/d) ^a^	8.6 (6.2, 11.8)	7.4 (5.5, 10.6)	−1.460	0.144
Omega-3 fatty acids (g/d) ^a^	1.1 (0.7, 1.7)	1.0 (0.7, 1.4)	−1.477	0.140
Omega-6 fatty acids (g/d) ^a^	7.0 (4.8, 10.1)	6.1 (4.5, 9.2)	−1.412	0.158

Data are shown as *n* (%) or median (25th and 75th percentile). E-DII, energy-adjusted dietary inflammatory index; MUFAs, monounsaturated fatty acids; PUFAs, polyunsaturated fatty acids; SD, sleep disturbance; SFAs, saturated fatty acids. ^a^ Mann–Whitney U test. ^b^ Chi-squared test.

**Table 3:** Association between categorical E-DII and sleep disturbance in patients with breast cancer (*n* = 302).

Variables	Model 1		Model 2		Model 3	
	OR (95% CI)	*p*	OR (95% CI)	*p*	OR (95% CI)	*p*
E-DII (categorical)						
T1 (−4.40, −0.90)	Reference	0.109 *	Reference	0.118 *	Reference	0.031 *
T2 (−0.89, 0.88)	0.73 (0.38, 1.38)		0.72 (0.38, 1.37)		0.88 (0.43, 1.81)	
T3 (0.89, 4.47)	1.81 (1.00, 3.27)		1.80 (0.99, 3.25)		2.34 (1.18, 4.62)	

Model 1 was unadjusted. Model 2 was adjusted for age and BMI. Model 3 was adjusted for age, BMI, physical activity level, education level, family monthly income, cancer stage, pain score, anxiety score, and depression score. 95% CI, 95% confidence interval; E-DII, energy-adjusted dietary inflammatory index; OR, odds ratio. * *p* value for trend derived using the median approach.

**Table 4:** Association between continuous E-DII or its components and sleep disturbance in patients with breast cancer (*n* = 302).

Variables	Model 1		Model 2		Model 3	
	OR (95% CI)	*p*	OR (95% CI)	*p*	OR (95% CI)	*p*
E-DII (continuous)	1.18 (1.02, 1.36)	0.026	1.17 (1.02, 1.35)	0.030	1.23 (1.04, 1.44)	0.014
Nutrients						
Dietary fiber	0.98 (0.94, 1.03)	0.467	0.99 (0.94, 1.03)	0.509	0.99 (0.96, 1.05)	0.959
Vitamin C	0.99 (0.99, 1.00)	0.001	0.99 (0.99, 1.00)	0.001	0.99 (0.99, 1.00)	0.015
Folate	0.99 (0.99, 1.00)	0.249	0.99 (0.99, 1.00)	0.253	0.99 (0.99, 1.00)	0.732
Niacin	0.96 (0.92, 1.01)	0.114	0.97 (0.92, 1.01)	0.130	0.97 (0.93, 1.02)	0.277
Magnesium	0.99 (0.99, 1.00)	0.019	0.99 (0.99, 1.00)	0.023	0.99 (0.99, 1.00)	0.190
Zinc	0.95 (0.89, 1.01)	0.119	0.95 (0.89, 1.01)	0.116	0.96 (0.90, 1.02)	0.306

Model 1 was unadjusted. Model 2 was adjusted for age and BMI. Model 3 was adjusted for age, BMI, physical activity level, education level, family monthly income, cancer stage, pain score, anxiety score, and depression score. 95% CI, 95% confidence interval; E-DII, energy-adjusted dietary inflammatory index; OR, odds ratio.

**Table 5:** Association between E-DII and plasma inflammatory biomarkers in patients with breast cancer (*n* = 103).

Variables	β	95% CI	*p*
IL-1β	0.066	0.03, 0.10	<0.001
IL-6	0.055	0.01, 0.10	0.021
IL-10	0.079	0.03, 0.13	<0.001
TNF-α	0.059	0.02, 0.10	0.003
CRP	0.050	0.01, 0.09	0.023

Data were shown as a beta coefficient and 95% confidence interval. Multiple linear regression was used after adjusting for age, BMI, physical activity level, education level, family monthly income, cancer stage, pain score, anxiety score, and depression score. 95% CI, 95% confidence interval; CRP, C-reactive protein; E-DII, energy-adjusted dietary inflammatory index; IL-1β, interleukin 1β; IL-6, interleukin 6; IL-10, interleukin 10; TNF-α, tumor necrosis factor α.

**Table 6:** Association between plasma inflammatory biomarkers and sleep disturbance in patients with breast cancer (*n* = 103).

Variables	OR	95% CI	*p*
IL-1β	1.01	1.00, 1.02	<0.001
IL-6	1.04	1.02, 1.16	<0.001
IL-10	1.03	1.01, 1.04	<0.001
TNF-α	1.01	1.00, 1.01	<0.001
CRP	1.26	1.10, 1.45	<0.001

Multivariate logistic regression was used after adjusting for age, BMI, physical activity level, education level, family monthly income, cancer stage, pain score, anxiety score, and depression score. 95% CI, 95% confidence interval; CRP, C-reactive protein; IL-1β, interleukin 1β; IL-6, interleukin 6; IL-10, interleukin 10; OR, odds ratio; TNF-α, tumor necrosis factor α.

**Table 7:** Statistical mediating effects of inflammatory biomarkers on the association between E-DII and sleep disturbance in patients with breast cancer (*n* = 103).

Mediators	ATE			ADE			ACME			*ρ*
	Estimate	95% CI	*p*	Estimate	95% CI	*p*	Estimate	95% CI	*p*	
IL-1β	0.088	0.023, 0.170	0.008	0.057	−0.004, 0.142	0.073	0.031	0.007, 0.066	0.004	0.3
IL-6	0.077	0.013, 0.169	0.040	0.050	−0.005, 0.135	0.077	0.027	0.001, 0.062	0.039	0.5
IL-10	0.082	0.010, 0.165	0.020	0.034	−0.026, 0.116	0.330	0.048	0.011, 0.096	0.004	0.4
TNF-α	0.076	0.020, 0.169	0.006	0.048	−0.005, 0.137	0.080	0.028	0.007, 0.063	0.004	0.4
CRP	0.079	0.013, 0.161	0.016	0.055	−0.005, 0.134	0.078	0.024	0.002, 0.057	0.032	0.4

The statistical mediating effect of IL-1β, IL-6, IL-10, TNF-α, and CRP between E-DII and sleep disturbance in patients with BC was calculated after adjusting for age, BMI, physical activity level, education level, family monthly income, cancer stage, pain score, anxiety score, and depression score. The 95% CI was computed from 5000 nonparametric bootstrap replicates. 95% CI, 95% confidence interval; ACME, average causal mediation effect; ADE, average direct effect; ATE, average total effect; CRP, C-reactive protein; E-DII, energy-adjusted dietary inflammatory index; IL-1β, interleukin 1β; IL-6, interleukin 6; IL-10, interleukin 10; TNF-α, tumor necrosis factor α.

## Data Availability

Data are available upon reasonable request from the corresponding author. The data contain information from human participants, and access is restricted to protect participant privacy and comply with ethical regulations.

## Supplementary Materials

The following supporting information can be downloaded at: https://www.mdpi.com/article/10.3390/nu17243889/s1, Figure S1: Path diagram of the mediation analysis of inflammatory biomarkers on the association between E-DII and sleep disturbance in patients with breast cancer. Table S1: Dietary nutrient intakes of breast cancer patients in different E-DII tertiles (*n* = 302). Table S2: Model fit information for logistic regression analyses of E-DII and its components with sleep disturbance. Table S3: Diagnostic of multicollinearity in categorical E-DII and sleep disturbance logistic regression models. Table S4: Diagnostic of multicollinearity in continuous E-DII and sleep disturbance logistic regression models. Table S5: Association between E-DII and continuous PQSI score in patients with breast cancer (*n* = 302). Table S6: Comparison of characteristics between participants with and without plasma inflammatory biomarkers.
