# Supplementary material for: The Potential Mediating Role of Inflammation on the Association Between Dietary Inflammatory Index and Sleep Disturbance Among Breast Cancer Patients: A Cross-Sectional Study
Source: Nutrients. 2025 Dec 12;17(24):3889. doi: 10.3390/nu17243889 (PMC12736269; doi:10.3390/nu17243889)
Supplement: Supplementary file 1 [file nutrients-17-03889-s001.zip › nutrients-3969335-supplementary.pdf]

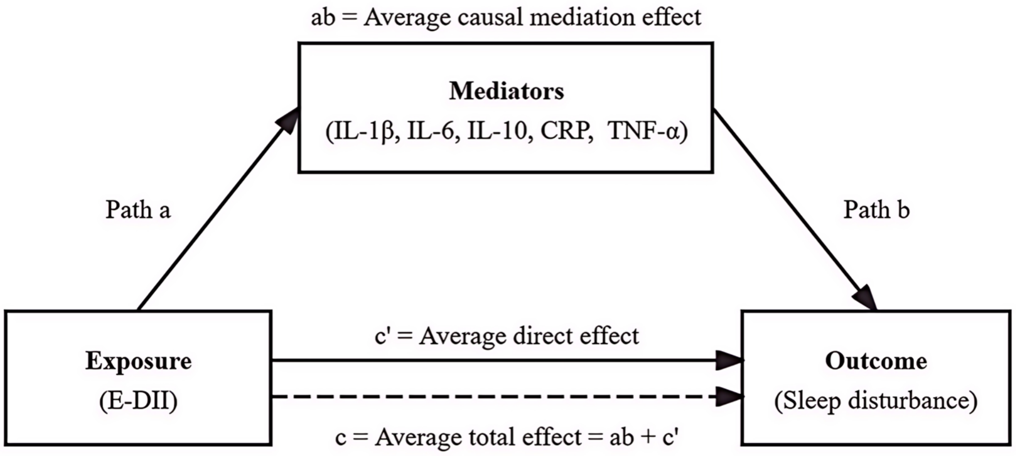

Figure.S1 Path diagram of the mediation analysis of inflammatory biomarkers on the association between E-DII and sleep disturbance in patients with breast cancer. CRP, C-reactive protein; E-DII, energy-adjusted dietary inflammatory index; IL-1β, interleukin 1β; IL-6, interleukin 6; IL-10, interleukin 10; TNF-α, tumor necrosis factor α.

Table S1. Dietary nutrient intakes of breast cancer patients in different E-DII tertiles (*n* = 302)

Variables	T1 (*n* = 101)	T2 (*n* = 101)	T3 (*n* = 100)	*F/H*	*P*
Energy (kcal/d) [a]	1243.58 ± 348.56	1267.38 ± 340.87	1439.37 ± 428.87	7.042	0.001
Protein (g/d) [b]	66.8 (51.3, 89.7)	56.9 (44.7, 75.9)	59.8 (44.9, 84.1)	6.882	0.032
Total fat (g/d) [b]	40.3 (31.8, 52.9)	49.9 (32.6, 64.5)	51.7 (35.4, 76.1)	13.569	0.001
Carbohydrate (g/d) [b]	141.3 (120.9, 166.3)	136.8 (106.9, 177.2)	160.1 (114.4, 207.1)	5.983	0.050
Dietary fiber (g/d) [b]	11.8 (8.7, 15.2)	8.7 (6.3, 11.2)	7.2 (5.0, 9.5)	55.039	< 0.001
Cholesterol (mg/d) [b]	635.0 (472.0, 804.5)	551.0 (319.5, 751.0)	522.0 (378.5, 729.3)	5.076	0.079
Vitamin A (μgRAE/d) [b]	548.0 (384.5, 716.5)	378.0 (291.5, 526.0)	339.5 (261.8, 476.0)	40.701	< 0.001
Vitamin B1 (mg/d) [b]	0.6 (0.5, 0.8)	0.6 (0.5, 0.8)	0.7 (0.5, 0.8)	0.659	0.719
Vitamin B2 (mg/d) [b]	1.1 (0.8, 1.6)	0.9 (0.7, 1.2)	0.9 (0.7, 1.2)	14.722	0.001
Vitamin B6 (mg/d) [b]	0.2 (0.1, 0.4)	0.2 (0.1, 0.2)	0.1 (0.0, 0.2)	47.411	< 0.001
Vitamin C (mg/d) [b]	137.2 (100.3, 217.0)	115.5 (76.5, 156.0)	69.0 (41.5, 105.0)	64.561	< 0.001
Vitamin D (μg/d) [b]	2.4 (1.0, 5.7)	1.6 (0.4, 4.9)	1.0 (0.0, 2.9)	13.280	0.001
Vitamin E (mg/d) [b]	18.3 (14.6, 24.3)	15.4 (11.0, 18.8)	13.3 (10.1, 17.3)	33.012	< 0.001
Folate (μg/d) [b]	143.7 (95.7, 245.4)	100.9 (67.6, 136.2)	68.6 (38.5, 107.8)	54.949	< 0.001
Niacin (mg/d) [b]	12.9 (9.4, 17.7)	12.1 (9.4, 15.0)	11.5 (7.8, 15.3)	3.861	0.145
Magnesium (mg/d) [b]	300.0 (246.0, 374.5)	236.0 (197.5, 308.0)	224.5 (175.0, 264.0)	44.875	< 0.001
Iron (mg/d) [b]	17.1 (14.8, 22.3)	14.6 (11.8, 19.1)	15.7 (12.2, 20.7)	10.471	0.005
Zinc (mg/d) [b]	10.3 (8.2, 12.8)	9.1 (6.7, 11.2)	8.3 (6.2, 10.9)	15.711	< 0001
Selenium (μg/d) [b]	60.3 (37.1, 86.9)	41.6 (29.9, 68.4)	45.2 (32.8, 61.0)	12.666	0.002
β-Carotene (ug/d) [b]	2459.7 (1393.7, 3926.3)	1356.8 (916.5, 2000.5)	820.2 (461.7, 1417.5)	74.117	< 0.001
SFA (g/d) [b]	11.6 (8.7, 16.5)	14.5 (11.0, 20.4)	15.5 (10.3, 23.0)	15.503	< 0.001
MUFA (g/d) [b]	15.1 (10.8, 20.9)	16.8 (11.4, 25.8)	18.4 (12.5, 27.9)	10.252	0.006
PUFA (g/d) [b]	9.2 (6.6, 13.3)	8.1 (5.8, 10.7)	7.6 (5.2, 10.7)	6.497	0.039
Omega-3 fatty acids (g/d) [b]	1.1 (0.9, 1.7)	1.0 (0.7, 1.4)	1.0 (0.6, 1.5)	4.973	0.083
Omega-6 fatty acids (g/d) [b]	8.2 (5.0, 11.3)	6.6 (4.8, 9.2)	6.2 (4.2, 9.3)	6.031	0.049

Data were shown as median (25th and 75th percentile) or mean ± standard deviation. [a] One-way ANOVA test. [b] Kruskal–Wallis test. E-DII, energy-adjusted dietary inflammatory index; E-DII tertile ranges: T1 (−4.40, −0.90), T2 (−0.89, 0.88), T3 (0.89, 4.47); MUFA, monounsaturated fatty acids; PUFA, polyunsaturated fatty acids; SFA, saturated fatty acids.

Table S2. Model fit information for logistic regression analyses of E-DII and its components with sleep disturbance

Variables	Model 1				Model 2				Model 3			
	R ^{2}	χ^2	***P****	***P*****	***R*** ^{2}	χ^2	***P****	***P*****	***R*** ^{2}	χ^2	***P****	***P*****
E-DII (categorical)	0.042	0.000	1.000	−	0.049	11.763	0.162	−	0.280	2.553	0.904	−
E-DII (continuous)	0.024	9.604	0.294	0.060	0.030	8.081	0.426	0.055	0.239	7.244	0.511	0.091
Dietary fiber	0.003	11.456	0.177	0.054	0.010	5.003	0.757	0.052	0.242	8.096	0.819	0.429
Vitamin C	0.057	9.664	0.289	0.274	0.062	9.841	0.276	0.287	0.266	5.918	0.656	0.136
Folate	0.007	14.018	0.081	0.051	0.014	9.282	0.319	0.059	0.218	6.312	0.612	0.055
Niacin	0.012	7.131	0.523	0.061	0.019	11.788	0.161	0.069	0.246	7.130	0.317	0.075
Magnesium	0.028	12.575	0.127	0.181	0.034	10.537	0.229	0.190	0.248	16.608	0.683	0.121
Zinc	0.013	8.980	0.344	0.088	0.020	13.029	0.111	0.106	0.246	11.857	0.237	0.141

Model 1 was unadjusted. Model 2 was adjusted for age and BMI. Model 3 was adjusted for age, BMI, physical activity level, education level, family monthly income, cancer stage, pain score, anxiety score, and depression score. E-DII, energy-adjusted dietary inflammatory index. R^2, Nagelkerke R^2. χ^2, *P**, Hosmer–Lemeshow goodness-of-fit test. *P***, Box-Tidwell test for linearity in the logit for continuous variables. −, Not applicable.

Table S3. Diagnostic of multicollinearity in categorical E-DII and sleep disturbance logistic regression models

Variables	VIF
E-DII (categorical)	1.015
Age	1.465
BMI	1.072
Physical activity level	1.205
Education level	1.533
Family monthly income	1.367
Cancer stage	1.049
Pain score	1.066
Anxiety score	1.908
Depression score	1.831

VIF, variance inflation factor; E-DII, energy-adjusted dietary inflammatory index; BMI, body mass index.

Table S4. Diagnostic of multicollinearity in continuous E-DII and sleep disturbance logistic regression models

Variables	VIF
E-DII (continuous)	1.015
Age	1.465
BMI	1.075
Physical activity level	1.206
Education level	1.530
Family monthly income	1.363
Cancer stage	1.049
Pain score	1.066
Anxiety score	1.907
Depression score	1.834

VIF, variance inflation factor; E-DII, energy-adjusted dietary inflammatory index; BMI, body mass index.

Table S5. Association between E-DII and continuous PQSI score in patients with breast cancer (*n* = 302)

**Variables**	**Model 1**		**Model 2**		**Model 3**	
	**β (95%CI)**	***P***	**β (95%CI)**	***P***	**β (95%CI)**	***P***
E-DII (continuous)	0.01 (0.00, 0.03)	0.130	0.01 (0.00, 0.03)	0.130	0.01 (0.00, 0.03)	0.150
E-DII (categorical)						
T1 (−4.40, −0.90)	Reference		Reference		Reference	
T2 (−0.89, 0.88)	0.00 (−0.08, 0.08)	0.970	0.00 (−0.08, 0.08)	0.950	0.02 (−0.05, 0.09)	0.600
T3 (0.89, 4.47)	0.05 (−0.03, 0.13)	0.210	0.05 (−0.03, 0.13)	0.200	0.05 (−0.02, 0.12)	0.170

Model 1 was unadjusted. Model 2 was adjusted for age and BMI. Model 3 was adjusted for age, BMI, physical activity level, education level, family monthly income, cancer stage, pain score, anxiety score, and depression score. 95% CI, 95% confidence interval; E-DII, energy-adjusted dietary inflammatory index.

Table S6. Comparison of characteristics between participants with and without plasma inflammatory biomarkers

Variables	Without inflammatory biomarkers (n = 199)	With inflammatory biomarkers (n = 103)	$t/Z/\chi^2$	P
E-DII (continuous) [b]	0.3 (−1.6, 1.5)	−0.3 (−1.3, 0.9)	−0.939	0.348
PSQI score [b]	6.0 (3.0, 8.0)	4.0 (3.0, 7.0)	−1.887	0.059
Age (y) [b]	53.46 ± 11.01	52.85 ± 11.55	−0.800	0.424
BMI (kg/m^2) [a]	24.07 ± 3.19	24.74 ± 3.37	1.468	0.142
Menopausal status, *n* (%) [d]				
Post-menopausal	126 (63.3)	58 (56.3)	1.121	0.290
Pre-menopausal	73 (36.7)	45 (43.7)		
Marital status, *n* (%) [d]				
Widowed/divorced/single	8 (4.0)	3 (2.9)	0.027	0.620
Married	191 (96.0)	100 (97.1)		
Education level, *n* (%) [c]				
Primary school or lower	45 (22.6)	20 (19.4)	0.709	0.871
Middle school	65 (32.7)	36 (35.0)		
High school/secondary	42 (21.1)	20 (19.4)		
Junior college or higher	47 (23.6)	27 (26.2)		
Employment, *n* (%) [c]				
Employed	46 (23.1)	38 (36.9)	2.798	0.247
Unemployed	46 (23.1)	16 (15.5)		
Retired	107 (53.8)	49 (47.6)		
History of night shift work, *n* (%) [d]				
No	155 (77.9)	89 (86.4)	2.649	0.104
Yes	44 (22.1)	14 (13.6)		
Residence, *n* (%) [c]				
Rural areas	41 (20.6)	28 (27.2)	3.203	0.202
Towns	19 (9.5)	5 (4.9)		
Urban areas	139 (69.8)	70 (68.0)		
Family monthly income (CNY), *n* (%) [c]				
< 3000	18 (9.0)	12 (11.7)	5.244	0.073
3000 ~ 5000	95 (47.7)	35 (34.0)		
> 5000	86 (43.2)	56 (54.4)		
Physical activity level, *n* (%) [c]				
Low	53 (26.6)	20 (19.4)	2.994	0.224
Moderate	140 (70.4)	77 (74.8)		
High	6 (3.0)	6 (5.8)		
Pain score [b]	0.0 (0.0, 1.0)	0.0 (0.0, 1.0)	−0.745	0.456
Anxiety score [b]	4.0 (2.0, 7.0)	4.0 (2.0, 6.0)	−0.619	0.536
Depression score [b]	3.0 (1.0, 6.0)	3.0 (1.0, 5.0)	−1.419	0.156

Table S6. *Cont.*

Variables	Without inflammatory biomarkers (n = 199)	With inflammatory biomarkers (n = 103)	$t/Z/\chi^2$	P
Presence of comorbidities, n (%) [d]				
No	138 (69.3)	65 (63.1)	0.933	0.334
Yes	61 (30.7)	38 (36.9)		
Smoking status, n (%) [d]				
Never	195 (98.0)	101 (98.1)	0.000	1.000
Former/current	4 (2.0)	2 (1.9)		
Drinking status, n (%) [d]				
Never	190 (95.5)	96 (93.2)	0. 193	0.572
Former/current	9 (4.5)	7 (6.8)		
Tea consumption status, n (%) [d]				
Never/former	174 (87.4)	91 (88.3)	0.002	0.965
Current	25 (12.6)	12 (11.7)		
Coffee consumption status, n (%) [d]				
Never/former	191 (96.0)	101 (98.1)	0.382	0.537
Current	8 (4.0)	2 (1.9)		
Chemotherapy cycle, n (%) [c]				
T0	51(25.6)	17 (16.5)	3.360	0.339
T1~T2	81 (40.7)	49 (47.6)		
T3~T4	44 (22.1)	24 (23.3)		
≥ T5	23 (11.6)	13 (12.6)		
Cancer stage, n (%) [c]				
Ⅰ	70 (35.2)	28 (27.2)	2.162	0.339
Ⅱ	109 (54.8)	65 (63.1)		
Ⅲ	20 (10.0)	10 (9.7)		
Surgery type, n (%) [d]				
Lumpectomy	72 (36.2)	41 (39.8)	0.242	0.623
Mastectomy	127 (63.8)	62 (60.2)		
Triple-negative breast cancer, n (%) [c]				
No	170 (85.4)	81 (78.6)	1.770	0.183
Yes	29 (14.6)	22 (21.4)		

Data are shown as n (%), median (25th and 75th percentiles), or mean ± standard deviation. BMI, body mass index; CNY, China yuan; SD, sleep disturbance. [a] Independent samples t-test. [b] Mann−Whitney U test. [c] Chi-squared test. [d] Chi-squared test with continuity correction.
